# Enteral bleeding in a former preterm girl with short bowel syndrome: Do not miss the diagnosis

**DOI:** 10.1111/jpc.15593

**Published:** 2021-05-28

**Authors:** Chiara Udina, Anna M C Galimberti, Matteo Bramuzzo, Grazia Di Leo, Egidio Barbi

**Affiliations:** ^1^ University of Trieste Trieste Italy; ^2^ Institute for Maternal and Child Health – IRCCS ‘Burlo Garofolo’ Trieste Italy

**Key Points**

1. Anastomotic ulceration (AU) is a late complication of bowel resection in infancy with a significant impact on patients' quality of life. Symptoms are usually insidious and the diagnosis is challenging: a high index of awareness is necessary not to miss the diagnosis.
2. The presence of iron deficiency anaemia refractory to oral iron supplementation in a child who underwent bowel resection in the neonatal period should always raise the suspicion of AU.
3. Standard endoscopic techniques are often negative for pathological findings; in these cases capsule endoscopy should be conducted.
4. Due to high recurrence after AU surgical resection, a medical approach should be the mainstay of therapy.

## Case Report

**Fig 1:**
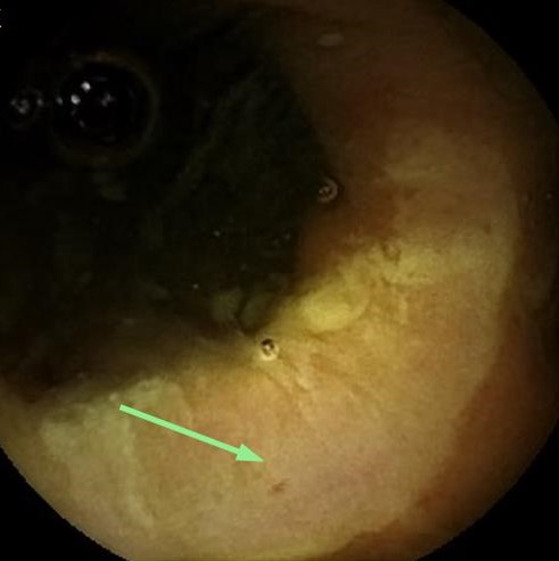
Large ileal post‐anastomotic circumferential ulcer revealed by capsule endoscopy.

A 9‐year‐old former preterm girl was referred to our attention for massive but self‐limiting enteral bleeding. Medical history was remarkable for neonatal necrotizing enterocolitis, subsequent wide ileal resection with ileocecal valve removal and jejuno colic anastomosis. Before enteral autonomy achievement, parenteral nutrition was required for the first 5 years of life due to short bowel syndrome (SBS). Mild chronic iron deficiency anaemia (IDA) was diagnosed 2 years before the admission and it was treated with oral iron supplementation with partial efficacy. Physical examination revealed sinus tachycardia (148 bpm) and a 3/6 systolic murmur at the apex. Laboratory tests showed severe microcytic anaemia (haemoglobin 4.9 g/dl, mean corpuscular volume 60 fl) with low ferritin (2.4 ng/L); folinic acid, vitamin B12, C‐reactive protein and erythrocyte sedimentation rate were within the normal range. After blood transfusion, colonoscopy and upper endoscopy were performed, both negative for pathological findings. Capsule endoscopy was therefore conducted, showing a large ileal post‐anastomotic circumferential ulcer (Fig. 1). Based on these findings, a diagnosis of anastomotic ulceration (AU) was made. After one intravenous dose of ferric carboxymaltose (15 mg/kg), the girl was discharged with oral iron supplementation, metronidazole and mesalamine. Haemoglobin increased gradually with achievement of a stable value (11 g/dl) after 6 months despite persistent faecal occult blood loss. At a 2‐year follow‐up, no recurrence of massive enteral bleeding occurred.

## Discussion

Anastomotic ulceration is a potential life‐threatening and under‐recognised condition that could complicate intestinal resection. Due to its rarity, the exact incidence in children is not well established, varying between 0.3%^1^ and 8%^2^ of resected patients. After the first report in 1988,^2^ only a few paediatric cases have been published, the largest series gathering 11 patients in 2014.^1^ Typically, affected children are former preterm neonates who underwent bowel resection soon after birth due to several underlying diseases, such as severe necrotizing enterocolitis, congenital malformations (atresia, gastroschisis, omphalocele), volvulus, and intussusception.

Symptoms of AU can occur up to 18 years after surgery and are usually insidious.^1^, ^3^ Main presenting feature is IDA refractory to oral iron supplementation. As SBS is a common comorbidity in these children, IDA is often misinterpreted as a consequence of malabsorption, leading to significant diagnostic delay. Acute massive enteral bleeding is less common but can require emergency treatment and can be potentially fatal. Lethargy, abdominal pain, growth retardation and hypoalbuminemia are also described.^1^, ^4^ Systemic inflammatory markers are negative and help differentiate AU and inflammatory bowel disease.

In the majority of patients, the aetiology of AU remains unknown. Several possible mechanisms have been proposed, but no one has been clearly demonstrated as a causative factor: exposure of ileal mucosa to colonic micro‐environment especially in case of ileocecal valve removal; local ischemia due to scar tissue at the anastomotic site; reaction to a foreign body in the form of surgical material; bile acid and pancreatic enzyme activity; abnormal motility and bacterial overgrowth.^4^

Because of previous extensive abdominal surgery and serial transverse enteroplasty, most patients have an unusual intestinal anatomy. Thus, the involved area may be difficult to visualise with standard endoscopic technique, contributing to diagnostic delay.^1^ Capsule endoscopy has proven to be a valid diagnostic tool^5^ that should be performed in all cases of presumptive AU, based on clinical and anamnestic characteristics despite normal upper endoscopy and colonoscopy. A high index of suspicion is, therefore, necessary to make a correct diagnosis.

Due to limited evidence base, no specific treatment is strongly recommended. Various therapies have been tried,^5^ including metronidazole, sulfasalazine, mesalamine, budesonide, antacid drugs, cholestyramine and, more recently, local treatment with argon‐plasma coagulation and platelet‐rich fibrin,^3^ with only limited success. Indeed, recurrences are frequent and many patients undergo repeated endoscopic or surgical treatments.^6^ Among 24 patients who underwent surgical ulcer resection, 67% had a relapse, which occurred 1 month to 3 years after the operation.^1^ Because of this high rate of early recurrence, in children who are often already affected by SBS, a medical approach should be the mainstay of therapy. On the basis of available data, first step management should consist of iron supplementation, decontaminant antibiotic treatment associations and topical anti‐inflammatory drugs, repeated over time to maintain remission. Due to the frequent need for intravenous iron supplementation, the use of ferric carboxymaltose could facilitate, as in our case, the management and quality of life of patients. Indeed, it provides controlled delivery of iron after a single high dose infusion that has proven to be effective and safe for chronic IDA from several causes.^7^ Surgery should be limited to life‐threatening bleedings or refractory symptomatic disease.

## References

[1] Charbit‐Henrion F , Chardot C , Ruemmele F *et al*. Anastomotic ulcerations after intestinal resection in infancy. J. Pediatr. Gastroenterol. Nutr. 2014; 59: 531–6. 24979478 10.1097/MPG.0000000000000472

[2] Parashar K , Kyawhla S , Booth IW , Buick RG , Corkery JJ . Ileocolic ulceration: A long‐term complication following ileocolic anastomosis. J. Pediatr. Surg. 1988; 23: 226–8. 3357138 10.1016/s0022-3468(88)80727-9

[3] Fusaro F , Tambucci R , Romeo E *et al*. Anastomotic ulcers in short bowel syndrome: New suggestions from a multidisciplinary approach. J. Pediatr. Surg. 2018; 53: 483–8. 28610705 10.1016/j.jpedsurg.2017.05.030

[4] Chari ST , Keate RF . Ileocolonic anastomotic ulcers: A case series and review of the literature. Am. J. Gastroenterol. 2000; 95: 1239–43. 10811334 10.1111/j.1572-0241.2000.02016.x

[5] Bass LM , Zimont J , Prozialeck J , Superina R , Cohran V . Intestinal anastomotic ulcers in children with short bowel syndrome and anemia detected by capsule endoscopy. J. Pediatr. Gastroenterol. Nutr. 2015; 61: 215–9. 25806676 10.1097/MPG.0000000000000778

[6] Hamilton AH , Beck JM , Wilson GM , Heggarty HJ , Puntis JW . Severe anaemia and ileocolic anastomotic ulceration. Arch. Dis. Child. 1992; 67: 1385–6. 1471894 10.1136/adc.67.11.1385 PMC1793781

[7] Ozsahin H , Schaeppi M , Bernimoulin M , Allard M , Guidard C , van den Ouweland F . Intravenous ferric carboxymaltose for iron deficiency anemia or iron deficiency without anemia after poor response to oral iron treatment: Benefits and risks in a cohort of 144 children and adolescents. Pediatr. Blood Cancer 2020; 67: e28614. 32729200 10.1002/pbc.28614

